# Supplementary material for: Atomic-scale observation of structural and electronic orders in the layered compound α-RuCl3
Source: Nat Commun. 2016 Dec 12;7:13774. doi: 10.1038/ncomms13774 (PMC5159869; doi:10.1038/ncomms13774)
Supplement: Supplementary Information — Supplementary Figures, Supplementary Table, Supplementary Notes and Supplementary References. [file ncomms13774-s1.pdf]

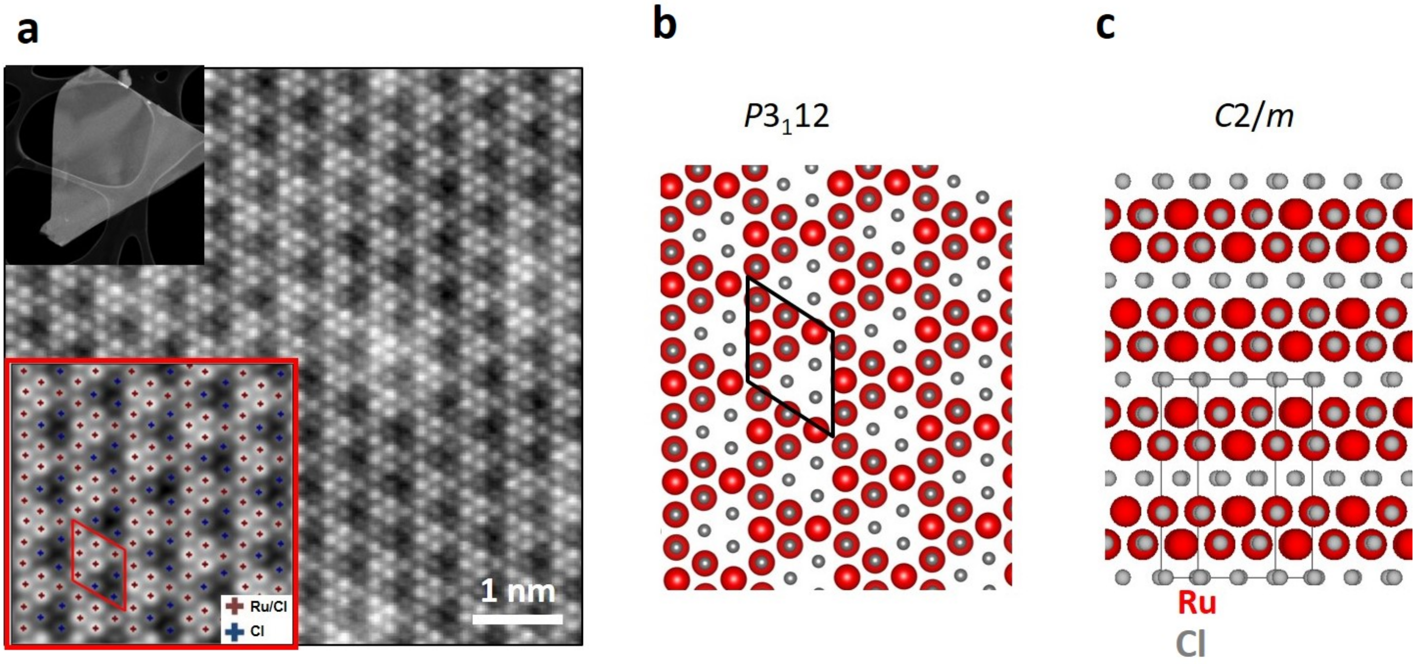

SUPPLEMENTARY FIGURE 1. Comparison of room temperature STEM data with proposed structural models. (a) Experimental STEM image; top left inset shows location of a flake on the grid. (b) $P3_1 12$ structural model. (c) $C2/m$ structural model. See also Supplementary Note 1.

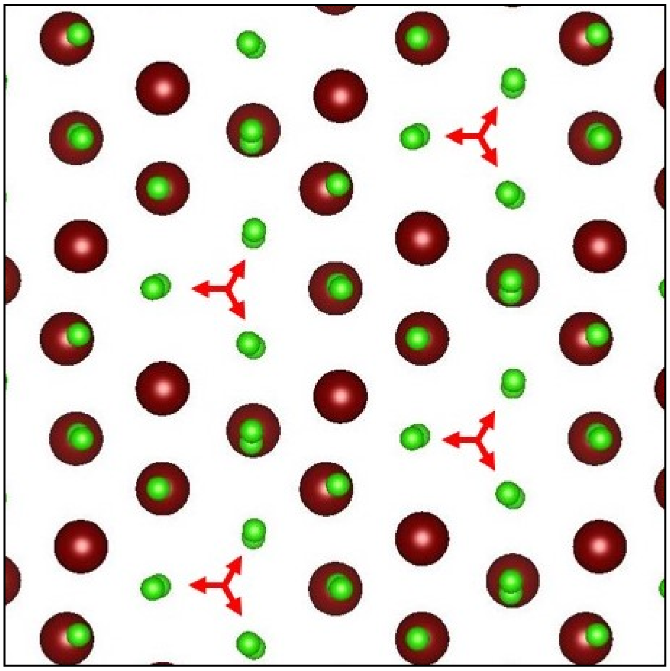

SIPPLEMENTARY FIGURE 2. Schematics of octahedral distortion found in neutron diffraction data on single crystals (top view) that leads to enlargement of Cl-Cl intercolumn distances (schematically denoted by red arrows); this agrees with STEM observations on exfoliated films. See also Supplementary Note 2.

a

b

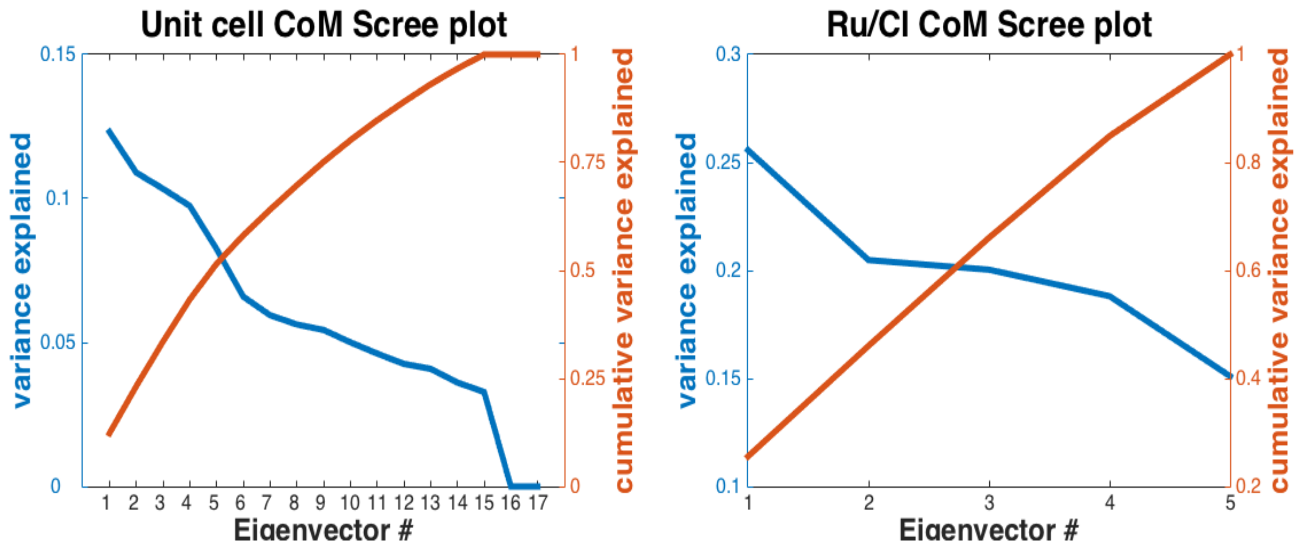

SUPPLEMENTARY FIGURE 3. PCA scree plots. (a) PCA scree plot for center of mass (CoM) of entire unit cell as origin and (b) center of mass of 6 Ru/Cl columns as origin.

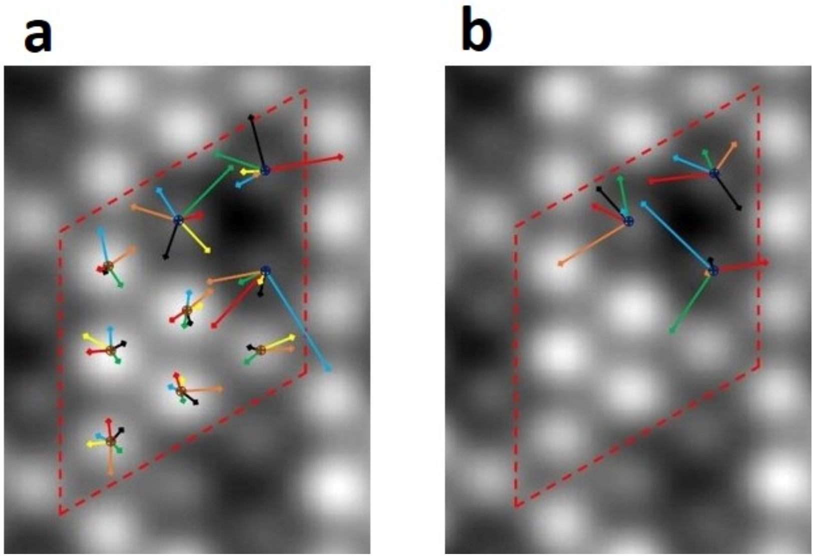

SUPPLEMENTARY FIGURE 4. PCA-derived first 6 normal modes of displacement for (a) center of mass of entire unit cell as origin; (b) center of mass of 6 Ru/Cl columns as origin.

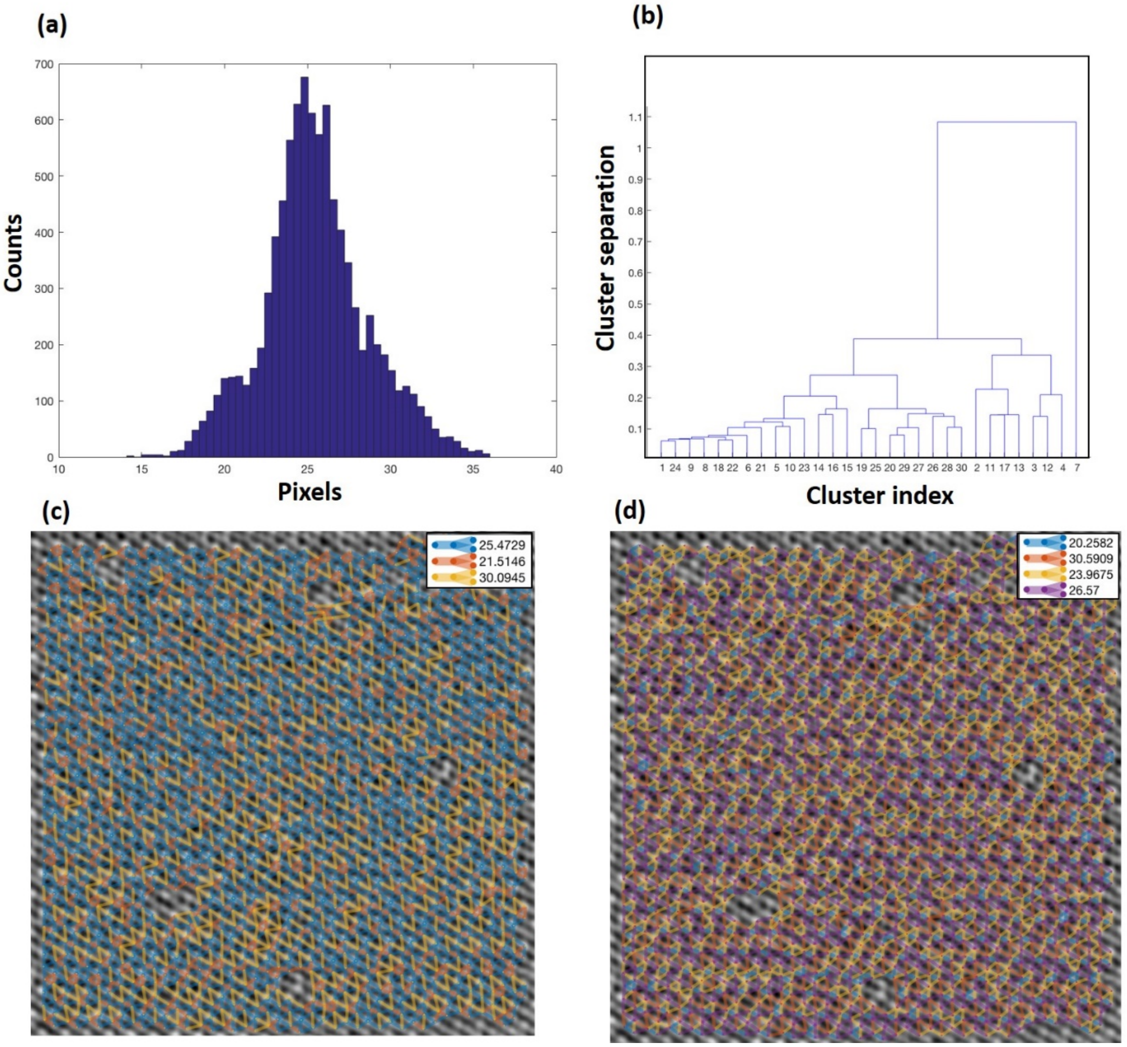

SUPPLEMENTARY FIGURE 5. The k-means clustering analysis. (a) Histogram of 6 nearest neighbor distances distribution from the STM image described in Fig. 4a of the main text (b) Dendogram plot of hierarchical binary cluster tree. (c,d) Real-space data for distribution of the nearest-neighbor distances for 3 clusters (c) and 4 clusters (d).

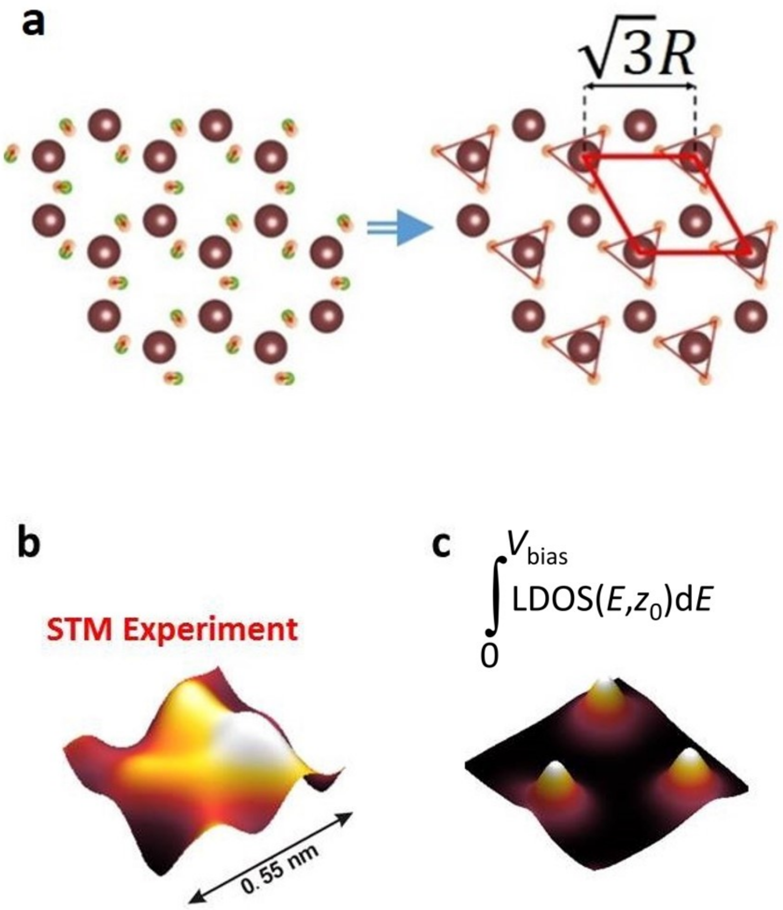

SUPPLEMENTARY FIGURE 6. Lattice distortion as a possible origin of observed charge order. (a) Left: Schematic view of the lateral component (projection onto *xy* plane) of Cl atoms displacement. The initial and final positions of atoms are denoted by green and orange balls, respectively. The actual displacements are exaggerated for a purpose of better visualization. Right: Projection of the resultant position of Cl atoms in the top layer that forms a structure with a $\sqrt{3}R \approx 600\mathrm{pm}$ periodicity. (b, c) Experimental (b) and DFT-simulated (c) STM images showing a distribution of charge density at 3 “top” Cl atoms in the unit cell at bias voltage V_{bias}=0.3V. No charge modulation was found within the current DFT scheme.

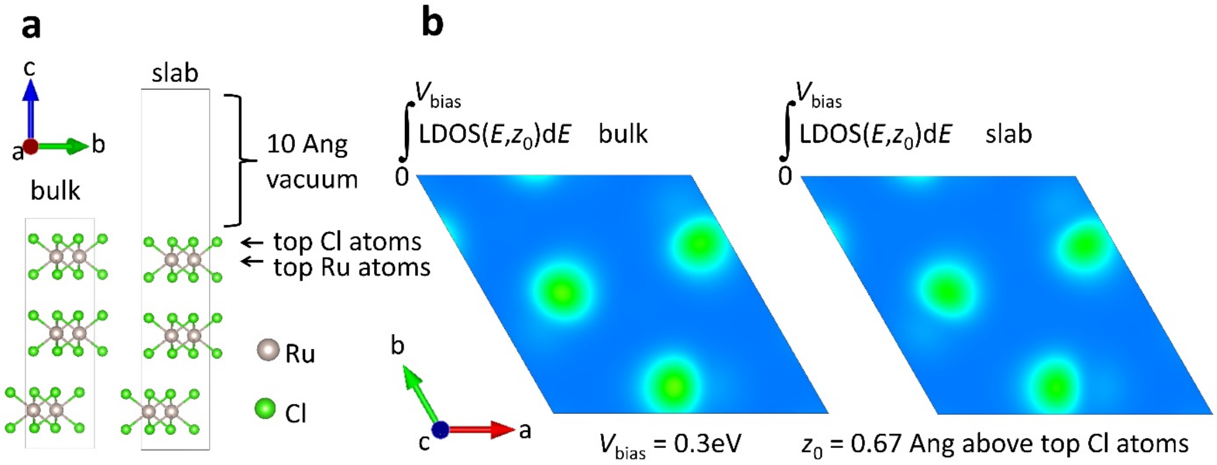

SUPPLEMENTARY FIGURE 7. DFT STM simulations for bulk and surface models. (a) Side view of bulk and slab unit cell. (b) Top view of energy integrated Local Density of States (LDOS) at 0.67 Ang above top Cl atoms from the bulk and the slab unit cell respectively.

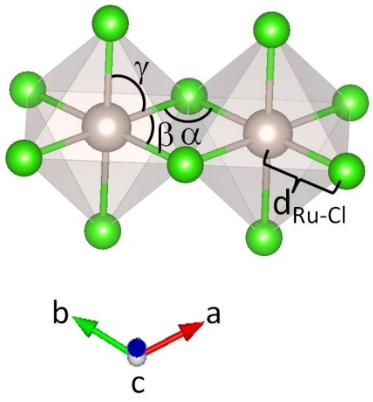

	Ru_6Cl_{18} bulk	top-layer Ru_6Cl_{18} slab
d_{Ru-Cl} (Ang)	2.362	2.363
α	92.48	92.44
β	87.52	87.56
γ	90.85	90.83

SUPPLEMENTARY FIGURE 8. Comparison of average structural parameters obtained from the bulk and the top-layer of the slab.

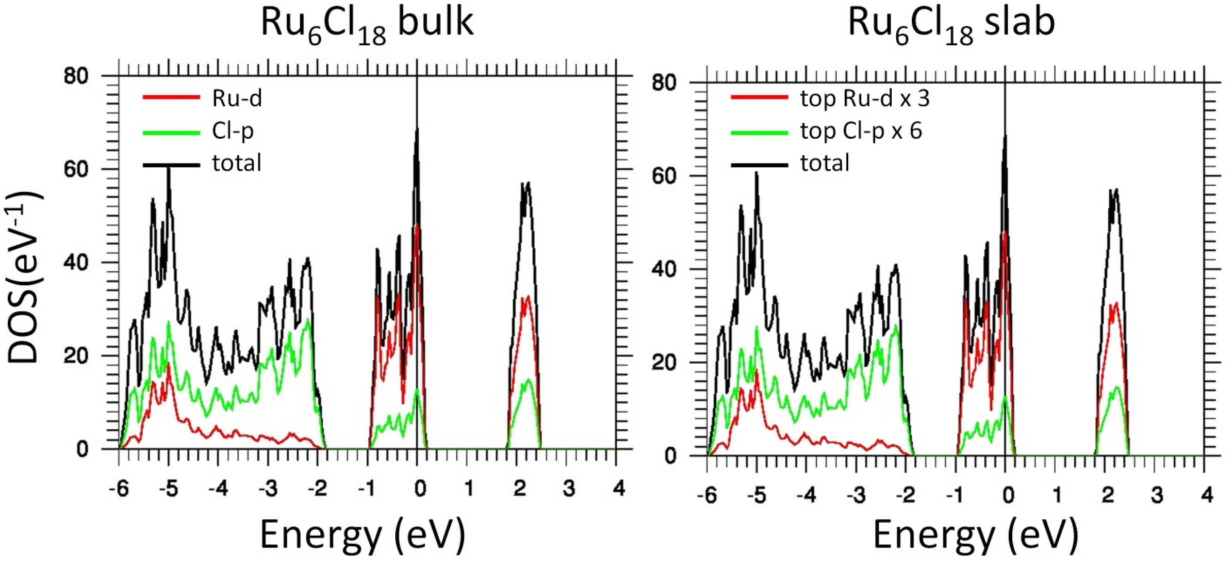

SUPPLEMENTARY FIGURE 9. Orbital resolved Density of States (DOS) from the bulk and the slab unit cell respectively.

Supplementary Table 1. The structure parameters of α-$RuCl_3$ measured at 200 K by single crystal neutron diffraction. The space group is $P3_1$, a=5.956 Å; c=17.054 Å, α=β=90°, γ=120°. *U* has units of Å^2.

Atom	Type	x	y	z	*U*
Ru1	Ru	0.44445	0.55555	0.03333	0.005
Ru2	Ru	0.07567	0.20533	0.36667	0.005
Cl3	Cl	0.13267	0.56633	0.00000	0.009
Cl4	Cl	0.12067	0.54833	0.46667	0.009
Cl5	Cl	0.08067	0.21233	0.66667	0.009
Cl6	Cl	0.11967	0.21333	0.13333	0.009
Cl7	Cl	0.40667	0.20233	0.00000	0.009
Cl8	Cl	0.42967	0.20533	0.46667	0.009

Supplementary Note 1

Comparison of STEM results with *P*3 and *C*2/*m* models

In $P3_112$ structure, each Ru/Cl column contains one Ru atom (Z_1=44) and two Cl atoms (Z_2=Z_3=17) per unit cell, whereas each Cl column contains two Cl atoms (Z_1=Z_2=17) per effective unit cell. As the intensity of 2-dimensional STEM image is roughly proportional to Z^2 and scales linear with a number of atoms in the column [1-3], the projection of the $P3_112$ structure on to the plane perpendicular to c*-axis would lead to a well-defined difference in contrast between three Cl columns and six Ru/Cl columns producing pattern with a periodic repetition of 6 brighter and 3 darker columns [see Supplementary Figure 1 (b)]. This agrees well with experimental observations. The situation, however, is different for *C*2/*m* structure. Here, when considering the projection on to the plane perpendicular to c*-axis, the Z^2 dependence of the intensity would produce a pattern with well-defined line features, in which every third line containing only Cl atomic columns appears substantially darker that the two other line originating from mixed Ru/Cl columns [see Supplementary Figure 1 (c)]. This does not fit the experimental observation.

Supplementary Note 2

Neutron diffraction measurements on single crystals

The single crystals neutron diffraction data reproduces well a lateral component of Cl cage distortion found in DFT and STEM (schematically shown with red arrows in Supplementary Figure 2; see also Supplementary Table 1 for details). It is noteworthy, however, that several other distortion components seen in neutron data, including a distortion in Ru sublattice, were not directly evident from STEM and DFT results. This can be explained by a relaxation of the structure upon exfoliation.

Supplementary Note 3

Computation of normal displacement modes from STEM image via principle component analysis (PCA).

PCA normal modes are computed from a set of atomic centers segmented into M periodic unit cells and represented by the matrix **N**. The rows of matrix **N** correspond to the unit cells and columns correspond to (x, y) coordinates of each of atoms within the studied system. The rows and columns represent "observations" and "variables", respectively. In the first analysis, (x, y) coordinates are computed for each atom within each unit cell with origin (0, 0) corresponding to the center of mass of all 9 atoms, resulting in 18 variables. In the second part of the analysis, we calculate the center of mass of Ru/Cl mixed columns, and (x, y) coordinates are only computed

for 3 pure Cl columns, resulting in only 6 variables. We run PCA to find statistical normal modes of displacements. PCA is used to convert the matrix **N** into a product of expansion coefficients a_{ik} and eigenmodes w_k [4, 5].

$$N_i(\omega_j) = a_{ik} w_k(\omega_j) \quad (1)$$

The eigenvectors are orthogonal and are arranged in descending order by variance. We do not perform mean subtraction, which makes the first eigenvector the average coordinates of atomic columns. Therefore in the main text, we start count from the second eigenvector. We note that PCA is a dimensionless type of analysis, which results in coordinates not corresponding to the coordinates we see in original data. In order to explore the variance in the atomic column positions we explore the 17 or 5 other eigenvectors in each case. Scree plots and percentage of the variance explained for both parts of the analysis are shown below in Supplementary Figure 3.

Selection of the number of eigenvectors explored is usually based upon the presence of a bend in the Scree plot. However, in the first case the estimation is somewhat obfuscated by eigenvectors 16 and 17 containing very little information, while in the second we have too few variables to disregard any. For the Ru/Cl center of mass analysis we have shown the plot for all 5 eigenvectors. For the unit cell center of mass we have shown the first 6 eigenvectors, as further exploration had revealed that eigenvectors 7 through 15 show mostly slight variations in the mixed Ru/Cl atomic positions, while the first 6 are most prominent in the pure Cl sublattice.

In Supplementary Figure 4a we show the data for center of mass of entire cell as origin for the first six eigenmodes. It is clear that the same trend is preserved, i.e. variation in geometry of Cl sublattice stronger than those in Ru sublattice. In Supplementary Figure 4b we show principal normal modes only for three Cl columns in the unit cell whose coordinates were recalculated with respect to the center of the mass of six Ru/Cl columns in each cell. Such analysis can provide greater details on the potential forms of distortion variation in Cl sublattice with respect to Ru-contained columns. The first eigenmode (blue arrow) shown in Supplementary Figure 4b can be described as a polar displacement mode of Cl columns, whereas the rest four eigenmodes can be assigned to a combination of shear-, stretch-, and rotation-like modes. Note that a small mismatch between the centers of the atomic cores of Cl atoms (less than 1.5 pm) calculated by DFT (and also found in neutron diffraction data) results in a fine splitting of each Cl column into 2 sub-columns. This splitting is beyond our experimental resolution and therefore the STEM views them as a single column, in which the center of the column corresponds to a “center of mass” of 2 sub-columns. Due to this fine structure of Cl columns, the presence of atomic vacancies in one of Cl sub-columns can shift a location of the center of mass. However, this alone cannot explain the complex distortion variation patterns observed in Supplementary Figure 4a and Supplementary Figure 4b. One possible additional contribution may originate from strain associated with a presence of Cl vacancies, which may induce tilts and rotations of $RuCl_6$ octahedral. Another component of distortional variation may arise from an interplay

between several alternative pathways of the $RuCl_6$ octahedra compression. Overall, our multivariate analysis implies a presence of complex local distortions in Cl ligand cages.

Supplementary Note 4

Identification of positions of electron density peaks in STM image

We first used FFT-filtered experimental STM images in order to acquire unit cell template and identify the coordinates of the centers of electron densities maxima within it. We then perform normalized cross correlation between the original image and the template and apply threshold of 0.70 to the resulting correlation surface in order to identify positions of probable centers of the unit cells.

Based on the coordinates in the template, for each potential center of the unit cell we first make a guess of one position. Using the proposed coordinate we perform intensity analysis within the 7-pixel radius of initial guess. We perform intensity threshold (with value of mean intensity of pixels in the selected area) and identify center of the largest resulting binary region. We assign this as the center of first (out of three) electronic density maxima for the unit cell.

We perform the same analysis for the 2 other protrusions associated with electronic density maxima, except at the final step we now check if newly found center is within 10 pixel distance to any other previously found center, in which case we do not record it. This alleviates the issue with identifying same centers twice due to low correlation surface threshold chosen to maximize number of identified unit cells.

After the refinement, we extract the sum of pixel intensities in a 9 by 9 pixel patch centered at each identified density center. We normalize the distribution to lie on the 0 to 1 interval.

Supplementary Note 5

Analysis of structural inhomogeneity in Cl sublattice from STM image of a surface monolayer.

The STM measurements reflect mainly the spatial distribution of the electron charge density near the Fermi level and are generally considered not as accurate as, for example, STEM measurements, for a detailed analysis of lattice structure. For example, the shift of charge density maxima with respect to Cl atomic cores may produce an apparent uniform distortion of individual hexagons of Cl atoms in the STM image of α-$RuCl_3$ [6]. On the other hand, as long as the electron density lattice is commensurate with the underlying atomic lattice, the *relative* distortions of the atomic positions in crystallographic lattice can be inferred from the STM data using statistical learning methods. Below we show a result of application of k-means clustering algorithm to high resolution STM image of Cl-terminated $RuCl_3$ surface. The k-means algorithm divides the dataset in a specified

number of optimally selected clusters, which define the groups of atoms with specific structure neighborhoods, so that the within-cluster sum of squares is minimized [5]:

$$\arg min \sum_{i=1}^{k} \sum_{x_j \epsilon S_i} \left\| x_j - \mu_i \right\|, \quad (2)$$

where μ_i is the mean of points in S_i. The dendogram and the resultant spatial distribution of nearest neighbor distances for the case of 3 and 4 clusters are shown in Supplementary Figure 5.

This analysis clearly reflects a non-uniform (inhomogeneous) distribution of the nearest-neighbor distances of electron density lattice points thus implying the distortion of the underlying Cl lattice itself. The latter is in the agreement with the structure inhomogeneity found in the STEM analysis discussed in the main text, but now it is demonstrated for a single surface layer of α-$RuCl_3$.

Note that several small areas in Supplementary Figure 5 were not identified in this analysis as their structure shows a larger deviation from average FFT unit cell than the rest of the structure.

Supplementary Note 6

(Uniform) lattice distortion as a possible origin of charge ordered state

In Supplementary Figure 6 we showed a projection of Cl atom distortion found in DFT onto the 2D surface plane. The symmetry of atomic distortions at the surface plane matches well with the symmetry of the *R*3 superlattice formed by the brightest spots in the STM image as illustrated in Supplementary Figure 6a. However, our DFT-simulated STM images performed on the structure with Cl distortion did not reproduce a charge imbalance at 3 Cl atoms in the unit cell, as is clearly seen from Supplementary Figure 6b and Supplementary Figure 6c.

Supplementary Note 7

Theoretical investigation of potential surface relaxation effects with PBE.

To theoretically investigate the possibility of surface relaxations in α-$RuCl_3$ we perform PBE calculations of a bulk and slab unit cell illustrated in Supplementary Figure 7. The bulk unit cell is taken to be the $P3_112$ unit cell (same as in the manuscript). The three layer slab is constructed from the bulk $P3_112$ unit cell by inserting 10 Ang of vacuum. Contrary to the calculations described in the manuscript, the lattice constants in the simulation described in this section are relaxed. Also approximate van der Waals corrections have been added in order to take into account the interactions between the $RuCl_3$ “sandwiches”, according to the DFT-D2 method of Grimme [7]. The other computational details are the same as described in the Method section of the manuscript.

As can be seen in Supplementary Figure 7 the integrated Local Density of States (LDOS) from the slab

and the bulk calculation are very similar and neither of them shows a reordering of the charge. Therefore surface relaxations do not seem to be a possible explanation of the experimentally observed charge order.

Supplementary Figure 8 compares the Ru-Cl bond distance (d_{Ru-Cl}) and three bond angles obtained from the bulk and the top-layer in the slab respectively. The small differences between the bulk and the surface results reflect the quasi 2D-ness of α-$RuCl_3$. The difference with results in the caption of Figure 3 of the main text stems from the fact that in the simulations described in this section the lattice parameters are relaxed instead being set to the experimental values.

Finally Supplementary Figure 9 compares the Density of States (DOS) from the Ru-d orbitals in bulk α-$RuCl_3$ and from the Ru-d orbitals in the surface layer of the three $RuCl_3$ layer slab with 10 Ang of vacuum described above. In particular we note that the Ru-t_{2g} band width in both cases are very similar (roughly within [-1.0, 0.2] eV) indicating that the overlaps between the Ru-t_{2g} (Wannier) orbitals in the bulk are very similar to those in the surface again reflecting the quasi 2D-ness of α-RuCl3.

Supplementary Note 8

Relevance of our data to quantum magnetism (extended discussion)

As explained below, the measurements presented in our manuscript should be valid in bulk samples and wide temperature regimes. This automatically leads to the relevance of our findings to the Kitaev physics. These specific points are as elucidated below:

Relevance of thin films and surface measurements to bulk α-$RuCl_3$ samples.

Based on the current experimental [8] and theoretical [9] understanding, it is reasonable to assume that the α-$RuCl_3$ can be treated as a quasi-2D material, in which each layer is nearly independent of the others. Taking into account the absence of dangling bonds, as well as the absence of alkali atomic layers above and below each $RuCl_3$ layer (in sharp contrast with iridates), we do not have any reason to believe that such quasi-2D system should develop any additional disorder *specific* to exfoliation/cleavage and reduction of the number of layers. This line of thinking is further supported by reports on similar layered compounds $TiCl_3$ and VCl_3 [10], as well as by conclusions of Weber *et al.* [11] that an in-plane structure of α-$RuCl_3$ is maintained in the exfoliation process. As shown in the Supplemntary Note 7, the surface relaxation effects can also be negated. Thus the modulations of structural and electronic orders reported in our study are very likely intrinsic to the bulk of the sample (i.e. our findings should be applicable to each of the quasi-isolated $RuCl_3$ layer inside a bulk sample).

Relevance to ‘Kitaev physics’.

a. We first note that the Kitaev interactions should be present within Mott ground state [12], an existence of which in our study was confirmed *via* STS measurements of local density of states in α-$RuCl_3$ surface monolayer.

The persistence of Mott state to high temperature also agrees with proposals put forward by Plumb et al. [13] and Zhou et al. [14].

b. In a 2D Kitaev QSL there is no phase transition into the paramagnetic state [15]. The spins are short-range correlated at low temperatures and as the temperature increases paramagnetic fluctuations overwhelm, however, there is no order parameter associated with this phase transition. This means that the same spin fluctuations which persist at low temperature should persist to, theoretically speaking, infinite temperature, although thermal fluctuations will make it harder to access them. This is already demonstrated in several papers and results, most notably *i)* Nasu et al. [16], where the bi-fermionic distribution is a fundamentally continuous evolution with temperature, up to very high temperatures close to room temperature, *ii)* ARPES measurements [14] that indicate the qualitatively same conclusions, and *iii)* the susceptibility measurements are definitely anisotropic to much above T_N [17-19] which shows that the magnetic interactions (K and J terms) responsible for the magnetic ground state and the magnetic Hamiltonian have formed at (and possibly above) room temperature, at which the current atomic-scale measurements were undertaken.

c. Finally, the peculiar charge order(s) we have found in our study will be a direct input for first-principle calculations to understand the role of such in determining the actual Hamiltonian of this material which has so far not been determined to any precision, especially since we believe it persists to low temperature and also in the bulk.

For all of the above reasons, it is natural to expect that our data captures much important information necessary to understand the quantum magnetism at low temperatures.

SUPPLEMENTARY REFERENCES:

[1] Pennycook, S. J. Z-contrast transmission electron microscopy: direct atomic imaging of materials. *Annu. Rev. Maler. Sci.* **22**, 171–195 (1992).

[2] Li, Z. Y. et al. Three-dimensional atomic-scale structure of size-selected gold nanoclusters. *Nature (London)* **451**, 46-48 (2008).

[3] LeBeau, J. M., Findlay, S. D., Allen, L. J., & Stemmer, S. Standardless Atom Counting in Scanning Transmission Electron Microscopy. *Nano Lett.* **10**, 4405–4408 (2010).

[4] Jesse, S. & Kalinin, S. V. Principal component and spatial correlation analysis of spectroscopic-imaging data in scanning probe microscopy. *Nanotechnology* **20**, 085714 (2009).

[5] Belianinov, A. He, Q. Kravchenko, M., Jesse, S. Borisevich, A. Kalinin, S. V. Identification of phases, symmetries and defects through local crystallography. *Nat. Commun*. **6**:7801 (2015).

[6] Ren, J.; Whangbo, M. H.; Bengel, H.; Magonov, S. N. Electronic origin of the low-symmetry scanning tunneling microscopy image of the layered transition-metal halide alpha-ruthenium(III) chloride. *The Journal of*

Physical Chemistry **97**, 4764-4768 (1993).
[7] Grimmie, S. Semiempirical GGA-type density functional constructed with a long range dispersion correction. *J. Comp. Chem.* **27,** 1787 (2006).
[8] Banerjee, A.; Bridges, C. A.; Yan, J. Q.; Aczel, A. A.; Li, L.; Stone, M. B.; Granroth, G. E.; Lumsden, M. D.; Yiu, Y.; Knolle, J.; Bhattacharjee, S.; Kovrizhin, D. L.; Moessner, R.; Tennant, D. A.; Mandrus, D. G.; Nagler, S. E. Proximate Kitaev quantum spin liquid behaviour in a honeycomb magnet. *Nat Mater* **15**, 733-740 (2016).
[9] Kim, H.-S.; Kee, H.-Y. Crystal structure and magnetism in α-$RuCl_3$: An ab-initio study. *Physical Review B* **93**, 155143 (2016).
[10] Zhou, Y.; Lu, H.; Zu, X.; Gao, F. Evidencing the existence of exciting half-metallicity in two-dimensional $TiCl_3$ and VCl_3 sheets. *Scientific Reports* **6**, 19407 (2016).
[11] Weber, D.; Schoop, L. M.; Duppel, V.; Lippmann, J. M.; Nuss, J.; Lotsch, B. V. Magnetic Properties of Restacked 2D Spin 1/2 honeycomb $RuCl_3$ Nanosheets. *Nano Letters* **16**, 3578-3584 (2016).
[12] Jackeli, G.; Khaliullin, G. Mott Insulators in the Strong Spin-Orbit Coupling Limit: From Heisenberg to a Quantum Compass and Kitaev Models. *Physical Review Letters* **102**, 017205 (2009).
[13] Plumb, K. W.; Clancy, J. P.; Sandilands, L. J.; Shankar, V. V.; Hu, Y. F.; Burch, K. S.; Kee, H.-Y.; Kim, Y.-J. α-$RuCl_3$: A spin-orbit assisted Mott insulator on a honeycomb lattice. *Physical Review B* **90**, 041112 (2014)
[14] Zhou, X.; Li, H.; Waugh, J.; Parham, S.; Kim, H.-S.; Sears, J.; Gomes, A.; Kee, H.-Y.; Kim, Y.-J.; Dessau, D. ARPES study of the Kitaev Candidate α-$RuCl_3$. Preprint at https://arxiv.org/abs/1603.02279 (2016).
[15] Nasu, J.; Udagawa, M.; Motome, Y. Vaporization of Kitaev Spin Liquids. *Physical Review Letters* **113**, 197205 (2014).
[16] Nasu, J.; Knolle, J.; Kovrizhin, D. L.; Motome, Y.; Moessner, R. Fermionic response from fractionalization in an insulating two-dimensional magnet. *Nat Phys* **2016**, *advance online publication*.
[17] Sears, J. A.; Songvilay, M.; Plumb, K. W.; Clancy, J. P.; Qiu, Y.; Zhao, Y.; Parshall, D.; Kim, Y.-J. Magnetic order in α-$RuCl_3$: A honeycomb-lattice quantum magnet with strong spin-orbit coupling. *Physical Review B* **91**, 144420 (2015).
[18] Banerjee, A.; Yan, J.; Knolle, J.; Bridges, C. A.; Stone, M. B.; Lumsden, M. D.; Mandrus, D. G.; Tennant, D. A.; Moessner, R.; Nagler, S. E. Neutron tomography of magnetic Majorana fermions in a proximate quantum spin liquid. Preprint at https://arxiv.org/abs/1609.00103 (2016).
[19] Kubota, Y.; Tanaka, H.; Ono, T.; Narumi, Y.; Kindo, K. Successive magnetic phase transitions in α-$RuCl_3$: XY-like frustrated magnet on the honeycomb lattice. *Physical Review B* **91**, 094422 (2015).
